# An End-to-End Natural Language Processing Application for Prediction of Medical Case Coding Complexity: Algorithm Development and Validation

**DOI:** 10.2196/38150

**Published:** 2023-01-19

**Authors:** He Ayu Xu, Bernard Maccari, Hervé Guillain, Julien Herzen, Fabio Agri, Jean Louis Raisaro

**Affiliations:** 1 Biomedical Data Science Center Lausanne University Hospital Lausanne Switzerland; 2 Unit8 SA Lausanne Switzerland; 3 Public Health Solutions Ltd Promasens Switzerland; 4 Department of Administration and Finance Lausanne University Hospital Lausanne Switzerland; 5 Department of Visceral Surgery Lausanne University Hospital Lausanne Switzerland

**Keywords:** medical coding, natural language processing, NLP, complexity prediction, prediction, decision support, machine learning, model, clinical decision support application, multimodal modeling, coding, algorithm, documentation, health record, electronic health record, EHR, development

## Abstract

**Background:**

Medical coding is the process that converts clinical documentation into standard medical codes. Codes are used for several key purposes in a hospital (eg, insurance reimbursement and performance analysis); therefore, their optimization is crucial. With the rapid growth of natural language processing technologies, several solutions based on artificial intelligence have been proposed to aid in medical coding by automatically suggesting relevant codes for clinical documents. However, their effectiveness is still limited to simple cases, and it is not yet clear how much value they can bring in improving coding efficiency and accuracy.

**Objective:**

This study aimed to bring more efficiency to the coding process to improve the selection of codes by medical coders. To achieve this, we developed an innovative multimodal machine learning–based solution that, instead of predicting codes, detects the degree of coding complexity before coding is performed. The notion of coding complexity was used to better dispatch work among medical coders to eventually minimize errors and improve throughput.

**Methods:**

To train and evaluate our approach, we collected 2060 cases rated by coders in terms of coding complexity from 1 (simplest) to 4 (most complex). We asked 2 expert coders to rate 3.01% (62/2060) of the cases as the gold standard. The agreements between experts were used as benchmarks for model evaluation. A case contains both clinical text and patient metadata from the hospital electronic health record. We extracted both text features and metadata features, then concatenated and fed them into several machine learning models. Finally, we selected 2 models. The first used cross-validated training on 1751 cases and testing on 309 cases aiming to assess the predictive power of the proposed approach and its generalizability. The second model was trained on 1998 cases and tested on the gold standard to validate the best model performance against human benchmarks.

**Results:**

Our first model achieved a macro–*F*_1_-score of 0.51 and an accuracy of 0.59 on classifying the 4-scale complexity. The model distinguished well between the simple (combined complexity 1-2) and complex (combined complexity 3-4) cases with a macro–*F*_1_-score of 0.65 and an accuracy of 0.71. Our second model achieved 61% agreement with experts’ ratings and a macro–*F*_1_-score of 0.62 on the gold standard, whereas the 2 experts had a 66% (41/62) agreement ratio with a macro–*F*_1_-score of 0.67.

**Conclusions:**

We propose a multimodal machine learning approach that leverages information from both clinical text and patient metadata to predict the complexity of coding a case in the precoding phase. By integrating this model into the hospital coding system, distribution of cases among coders can be done automatically with performance comparable with that of human expert coders, thus improving coding efficiency and accuracy at scale.

## Introduction

### Background

Medical coding [1] is the translation of health care diagnoses and procedures into standard diagnosis and procedure codes using medical classifications and controlled terminologies. It is a strategic activity for funding hospitals and, therefore, its optimization is a priority in health care systems under financial pressure. In many countries worldwide, including Switzerland, hospital funding is based on the so-called *Prospective Payment System* [2,3] mechanism. In the Swiss Prospective Payment System, for example, inpatient stays are assigned to diagnosis-related groups [4] according to diagnosis and procedure codes derived from medical documentation, and each hospital stay is paid according to the diagnosis-related group to which it is assigned. Therefore, medical coding is closely linked, on the one hand, to medical documentation, and on the other hand, to hospital revenues. In addition to establishing reimbursement claims, medical codes are used for several other goals, such as setting budgets for planned hospitalizations or evaluating the quality of care by means of indicators such as complication rates after surgery.

The diagnosis and procedure codes of a specific case (ie, inpatient stay) are derived from clinical documentation such as discharge letters, surgical reports, physicians’ and nurses’ notes, and laboratory and radiologic results. The International Statistical Classification of Diseases and Related Health Problems, 10th Revision (ICD-10) [5], is usually used for coding diagnoses, whereas the classification system used to code procedures can vary from country to country [6].

Codes are manually entered into a hospital information system. In Switzerland, there are >200 coding rules that govern code entry and must be applied by medical coders. The latter are health care professionals who have undergone specific training for this purpose. However, despite training, medical coding remains a complex, quickly evolving, time-consuming, and error-prone task. In our tertiary academic medical center, medical coding staff have been divided into specialty teams since 2018. In a batch of cases, 50% are distributed to a “common pot,” and the other 50% are distributed to the corresponding specialty teams of medical coders. The cases in the “common pot” are distributed randomly to each team. A higher percentage of cases for the specialty teams is not envisaged for 3 reasons. First, it could lead to a loss of knowledge in general coding. Second, it could cause boredom for medical coders. Third, it will not always be possible to guarantee a sufficient number of cases for certain teams. Thus, a way to increase the efficiency of the current distribution of work without going toward a counterproductive overspecialization [7] is to force cases requiring high expertise to be assigned to experienced and specialist coders. This approach is only possible by detecting the complexity of the cases in advance before they are distributed and coded.

In recent years, artificial intelligence (AI) methods have been increasingly proposed to improve the efficiency and accuracy of medical coding. Their main goal has been to support medical coders in finding the most appropriate diagnosis and procedure codes for a given medical documentation. Conventional models, deep learning models such as convolutional neural networks and long short-term memory, and transformers have been trained and tested on automatic coding tasks using publicly available data sets in English [8-13]. Recently, this work has also been expanded to non-English corpora such as the French corpus [14,15]. In addition to the academic approach, commercial software for automatic coding has also been developed and introduced to the market. For example, commercial software such as ID SUISSE [16] applies rule-based algorithms to perform automatic coding. Their principle is to use a prebuilt dictionary of ICD-10 codes and their text labels, try to find clinical text that matches the labels, and then convert the text to ICD-10 codes. More recent tools such as Collective Thinking [17] and 360 Encompass (3M) [18] have improved the rule-based algorithms with machine learning (ML) techniques. Finally, solutions such as Sumex [19] rely on statistical methods to analyze the distributions and combinations of ICD-10 codes to identify possible inconsistencies in the coding patterns.

Despite the increasing number of available solutions, the effectiveness of automatic coding is still limited. Among the best-performing ML models, although precision can reach approximately 75%, the macro–*F*_1_-score could only achieve 10% to 12% [12,20,21]. The results indicate that even the best models can only capture a small portion of medical codes from free text. Therefore, the improvement of medical coding using AI-assisted strategies remains an open challenge (Kaur R, unpublished data, July 2021).

### Objectives

The purpose of our study was not to find a way to predict ICD-10 codes from medical records. Instead, it was to improve coding quality and efficiency by predicting coding complexity before the coding process. Our primary objective was to bring more efficiency to the coding process to improve the quality of coding by medical coders, and the means to achieve this is an innovative solution using ML. The innovation is to use ML to detect complexity, which is then used to better dispatch the work among medical coders. To the best of our knowledge, this approach has never been used before. It allows for a more efficient distribution of cases according to coders’ abilities and experience. As such, we will be able to minimize potential human errors because of random assignment and uneven distributions of coding expertise within hospitals’ coding divisions or units. Eventually, by knowing the coding complexity up front, simple cases can be assigned to beginners or nonspecialist coders or AI-assisted systems to maximize their utility while complex cases for which AI-assisted tools are still inefficient are assigned to coding specialists or at least to experienced medical coders.

Depending on the amount of clinical documentation to be examined and other factors such as the length of stay or the diversity of medical specialists involved in the treatment of a patient, coding a case may be a simple or a really complex task. Once a case has been coded, it is typically easy for the person who has done so to classify the case into a complexity level, which represents the complexity of the coding activity. However, predicting the complexity level of a case up front is very time-consuming for a human coder as it requires a deep analysis of the entire documentation, which eventually is equivalent to conducting the coding process directly.

To predict the complexity of a coding task in the precoding phase in an automatic way, we used advanced natural language processing (NLP) techniques to analyze clinical texts and extract features that are predictive of the complexity of cases. We proposed an end-to-end approach that integrates the NLP and ML model into the hospital clinical data warehouse and end-user coding system. Our NLP and ML model predicts case complexity with an accuracy comparable with that achieved by expert human coders. Its beta version is currently under deployment at Lausanne University Hospital. To the best of our knowledge, we are the first to propose and develop this innovative approach.

The remainder of the paper is organized as follows. The application details are presented in the *Methods* section, and the performance and analysis are presented in the *Results* section. In the *Discussion* section, we discuss the values and importance of our application as well as the use of NLP in health care.

## Methods

### Ethics Approval

The Cantonal Ethics Commission for research on human beings of Canton Vaud granted a full waiver for this study given the its retrospective and quality assurance nature under Req-2022-00677.

### Overview

**Figure 1:**
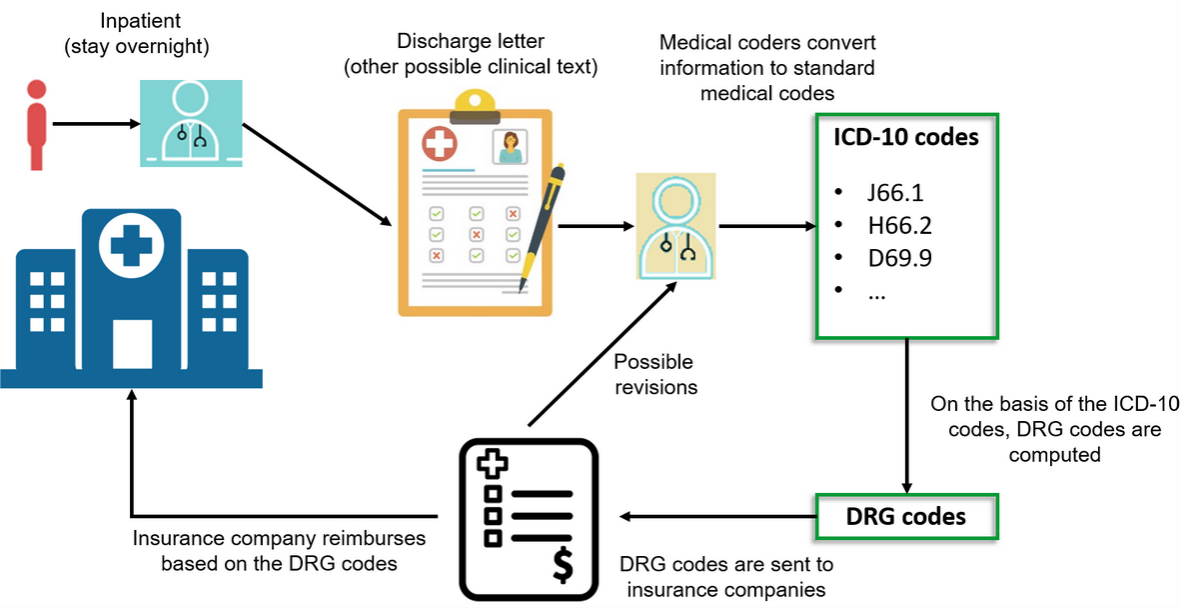
The general coding procedure in hospitals. DRG: diagnosis-related group; ICD-10: International Statistical Classification of Diseases and Related Health Problems, 10th Revision.

**Figure 2:**
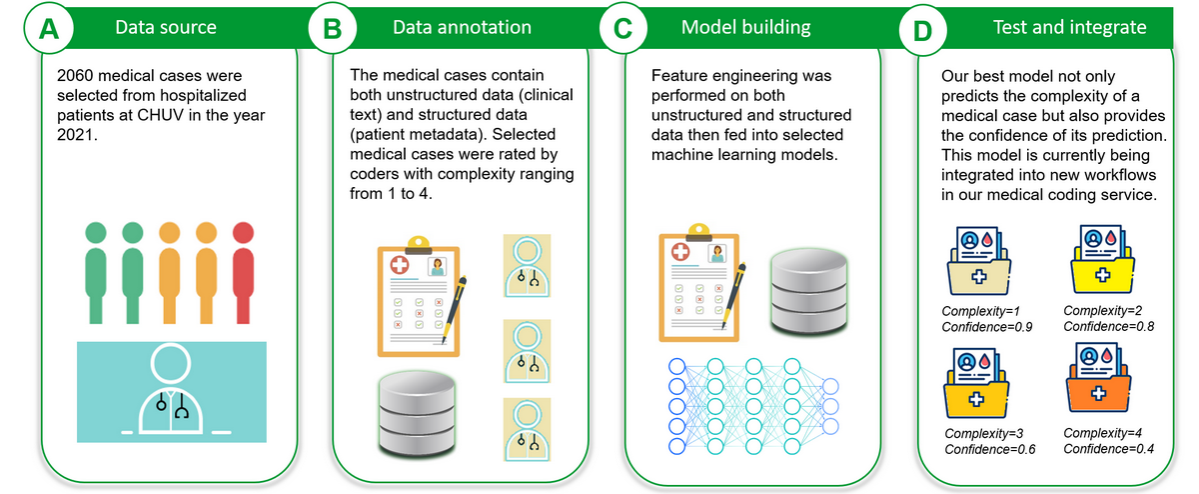
Workflow of this study. (A) We extracted 2060 cases from the clinical data warehouse at Lausanne University Hospital (CHUV). The cases are rated by coders (B) with complexity ranging from 1 (simplest) to 4 (most complex). (C) We performed feature engineering and trained models on the labeled cases. (D) The final model can produce both predictions of the complexity and its confidence in the predictions.

**Figure 3:**
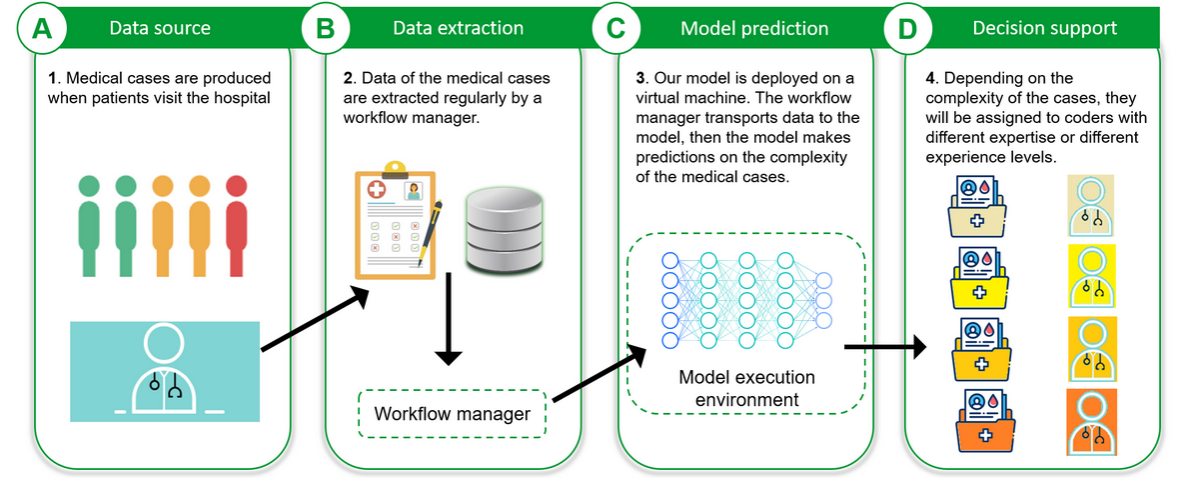
Integration of our model into the coding service. (A) When an inpatient visits the hospital and their medical case has been produced, the clinical text and patient metadata are stored in our clinical data warehouse. (B) A workflow manager will extract new medical cases regularly and send the data to our model. (C) Our model is containerized and deployed to an execution environment, where it performs the prediction for received cases. (D) Model predictions, together with the confidence of the predictions, are presented to the end users through a user interface to support task distribution in the coding service.

We describe a typical medical coding workflow in Figure 1. After an inpatient (patient who is hospitalized overnight) is treated in the hospital, a discharge letter is produced. Medical coders analyze the diagnosis in the discharge letter and translate the diagnosis into International Statistical Classification of Diseases and Related Health Problems, 10th Revision (ICD-10) codes. Sometimes the coders need to refer to other clinical documents (eg, intervention protocol and laboratory reports) to translate the information accurately. The diagnosis-related group codes are computed based on the ICD-10 codes and are sent to the insurance companies for billing. The insurance companies reimburse the bills to the hospital based on the received diagnosis-related group codes. If the insurance companies find mistakes in the codes, they ask for revisions from the coding service. We provide an overview of our decision support system in Figure 2 and describe its integration into the hospital information system in Figure 3.

### Definition of Complexity

We use the term “coding complexity” to characterize the time and expertise required of medical coders to assign diagnostic codes to medical cases.

Expertise can be defined as the level of experience, medical knowledge, and mastery of coding rules. Therefore, a medical case can be complex by applying many coding rules without being difficult but increasing the possibility of attention errors. Other cases may be complex and difficult because of the medical knowledge they require for proper coding. Therefore, complexity was the measure chosen to categorize the cases.

**Figure 4:**
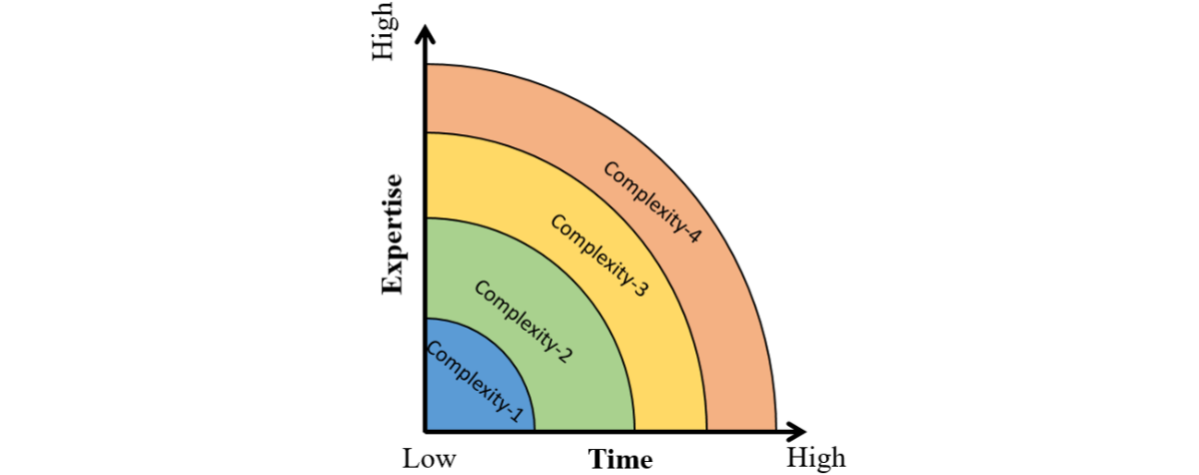
Intuitive representation of coding complexity regarding the time and expertise required of a coder.

If coding a medical case does not require much time and deep expertise, the coding complexity is low (level 1; Figure 4). Conversely, if coding a medical case requires a lot of time and deep expertise, the coding complexity is high (level 4; Figure 4).

Coding complexity, similar to pain or satisfaction, is a subjective quantity. A potential objective way of defining coding complexity can be provided by the automatic coding models. By passing the medical cases through automatic coding models and manually examining the confidence score and the completion and accuracy of ICD-10 code predictions, we could divide the cases into simple and complex groups. However, owing to the limited performance (ie, the very low recall score) of current automatic coding models regardless of language [12,20,21], this approach will not bring much value to our situation. Furthermore, if coding complexity could be measured using simple objective data (eg, similar to blood pressure), our multimodal modeling approach would be useless. Thus, in this study, our definition of coding complexity will focus on the subjective ratings provided by medical coders, aiming to minimize subjectivity by using ML approaches and to predict the subjective scores of complexity.

To train our ML model, we extracted 2060 medical cases from hospitalized patients (inpatients) in 2021. We organized 2 annotation phases, each lasting 1 week, for 28 coders to rate the cases’ complexity. During each annotation phase, the coders rated the complexity of the given cases based on an evaluation grid (Figure 4).

### Data Collection and Preprocessing

#### Data Source and Data Annotation

A medical case contains 2 types of data: a patient’s medical dossier and patient metadata (Textbox 1). We collected 2060 cases in total from the annotation phases. We note that the coding team at our hospital consisted of coders specialized in different medical domains. Hence, during annotation, we also kept track of whether a case was coded by a specialist. For example, if the responsible unit for a case was the internal medicine unit and the coder who coded this case was specialized in cardiology cases, the case was considered as not coded by its specialist coder.

Of the 2060 collected cases, 1998 (96.99%) were annotated by 28 medical coders, with each case coded by only 1 coder to maximize the size of the annotation set. As different medical coders may have different perceptions of the complexity of the same case, we evaluated the interrater reliability by asking 2 expert coders to code another 3.01% (62/2060) of cases. These 62 cases also represented our gold standard to create benchmarks for the models’ performance. For case selection, we first trained several models using the 1998 cases; then used the best model’s prediction to predict the complexity of several cases from our data warehouse; and, finally, randomly selected 62 out of the predicted cases while making sure that the complexity distribution of these 62 cases followed the same complexity distribution as the annotated data set. Each of the 62 cases was rated by each of the expert coders, and they were considered specialists for all cases. These 62 cases are referred to as the gold-standard set.

**Data collected for training and testing the model.**

- Patient metadata: responsible medical service, number of movements between medical services, age, gender, civil status, whether the patient was deceased, length of stay, and whether the case was coded by a specialist
- Medical dossier: discharge letter of each service, operating procedure, intervention reports, and death letter

#### Metadata Preprocessing

**Figure 5:**
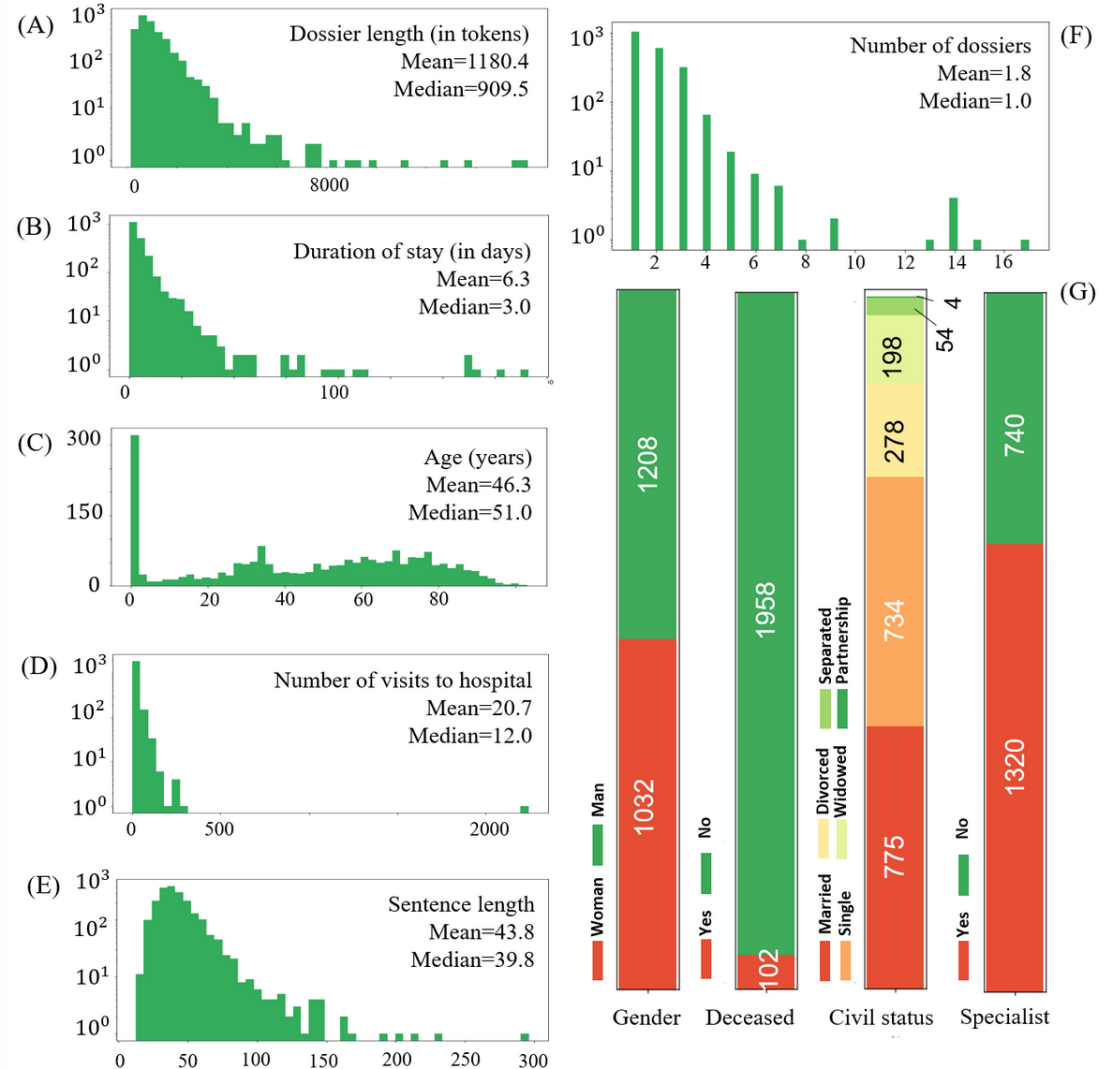
An overview of the distribution of patient metadata per stay. Document length and sentence length are counted in terms of tokens (words and punctuation marks). The distributions on A, B, D, and E are heavily skewed. Note that the distributions on A, B, D, E, and F are log-scaled. The rightmost column of G is deduced from the coder’s team specializations. The age=0 cases in C represent newborn cases.

The missing patients’ metadata were imputed based on the nature of the data. For numerical values such as age and length of stay, the missing values were imputed with the median of the existing values because of their skewed distributions (Figure 5). For categorical values such as gender and civil status, the missing values were imputed with the mode of existing values.

#### Text Data Preprocessing

We tested both classic term frequency-inverse document frequency (TF-IDF)–based text encoding and ML-based text encoding, and different text preprocessing steps were applied accordingly. For TF-IDF text encoding, we first tokenized the text; then removed the stop words; and, finally, replaced the entities with their entity type. The second and third steps were used to reduce the noise and increase the frequency of important words to provide a better signal for the model. An example of processed text is presented in Textbox 2.

For ML-based text encoding such as fastText (Facebook AI Research lab) and transformers, no preprocessing was applied.

**An example of text preprocessing results.**

- *Original text: Le patient susnommé a séjourné dans notre service du 01.02 au 03.02, date de son retour à domicile*.
- *Processed text: [“patient,” “susnommé,” “séjourné,” “service,” “<date>,” “<date>,” “date,” “domicile,” “.”]*

### Model Design

#### Overview

The overall approach of the model design was as follows. First, we extracted features from the preprocessed metadata and text data. Second, we tested 2 modeling approaches: framing the problem as a classification problem or as a regression problem. On the basis of the modeling approach, we used different metrics to evaluate the model performance.

#### Feature Engineering

As the values for the patients’ metadata have different scales, we applied standardization (*z* score) to the numerical data and one-hot encoding to the categorical data.

To extract features from free text, we used 2 methods: TF-IDF and word embeddings.

TF-IDF provides a numerical weight of how important a word is to a collection of documents (Multimedia Appendix 1). We tested 2 configurations of the TF-IDF method: using the top 10,000 frequent terms or using the top 1000 frequent terms. We found that, using the top 10,000 frequent terms, the models performed better than using only the top 1000 frequent terms. Thus, in the following sections, we only report the results from the TF-IDF vector using the top 10,000 frequent terms.

Word embeddings provide the vectorized representation of a word based on the context in which it appears. We tested three types of word embeddings: (1) word2vec [22,23] embeddings trained on 2.5 million clinical texts (12 GB) collected from the hospital’s clinical data warehouse; (2) the pooled output (CLS tokens) of the state-of-the-art French-language transformer model French-Language Understanding via Bidirectional Encoder Representations from Transformers (FlauBERT) [24], which was pretrained on 71 GB of French text collected from the internet; (3) the fastText supervised approach [25] with embeddings initialized with the pretrained word2vec embeddings of (1)—we tested fastText as it provided the subword approach that could reduce the impact of the out-of-vocabulary (OOV) issue. A detailed analysis of OOV for this study is provided in Multimedia Appendix 1.

Textbox 3 shows the sizes of the vectors extracted using the different methods. The detailed conversion methods are presented in Multimedia Appendix 1.

**Vector sizes of text feature engineering.**

- Term frequency-inverse document frequency (vectors were extracted using scikit-learn [version 1.0.1]): 10,000
- fastText (initialized with customized embedding; fastText embeddings were extracted using fastText [version 0.9.2; Facebook Artificial Intelligence Research lab]): 100
- word2vec (customized; word2vec embeddings were trained using Gensim [version 4.0.0; RARE Technologies, Ltd]): 100
- French-Language Understanding via Bidirectional Encoder Representations from Transformers (FlauBERT; the FlauBERT embeddings and fine-tuned model were implemented using Hugging Face [version 4.17.0; Hugging Face, Inc]): 768

#### Model Architecture

**Figure 6:**
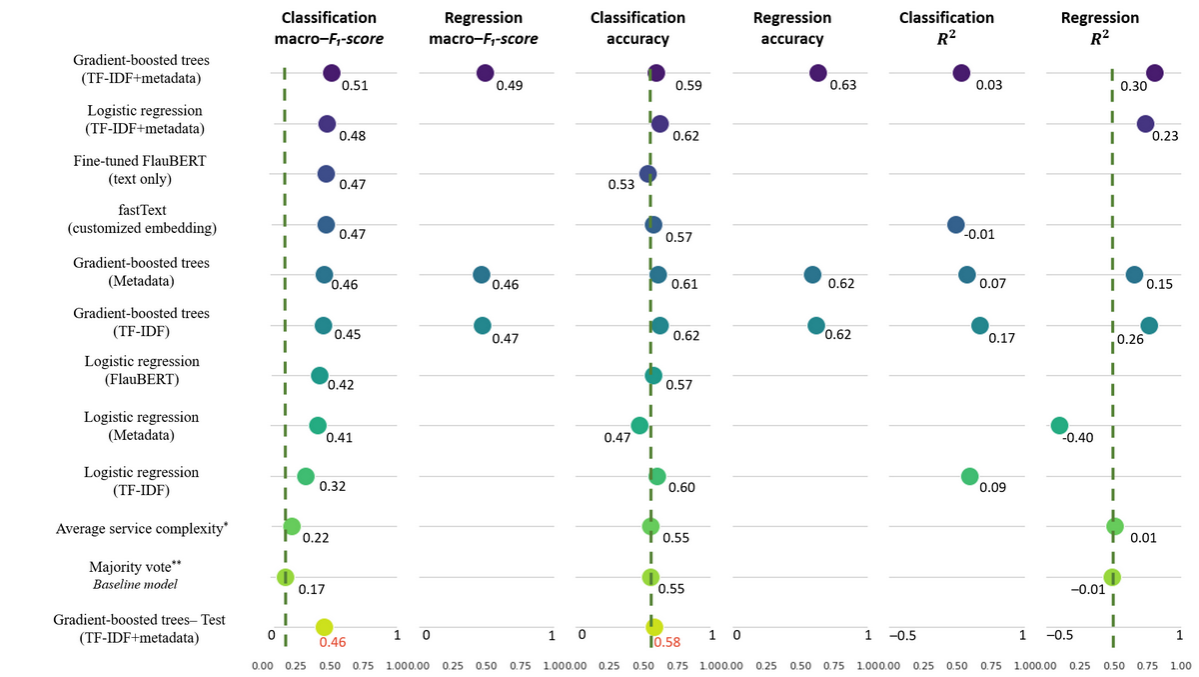
Comparison of performance using different models and input features on the 5-fold–cross-validated training data set (1751 cases) and the best model performance on the test set (309 cases). Dashed vertical lines represent the baseline model results. Models are ranked based on the classification macro–F1-score in the figure. *Average per service: for a given case in a given service, it always predicts the average complexity of cases in this service. A total of 29 services have an average complexity of 2, a total of 5 services have an average complexity of 3, and a total of 1 service has an average complexity of 1. **Majority vote: always predicts the majority class (in our case, complexity 2) and serves as a baseline for model prediction performance. FlauBERT: French-Language Understanding via Bidirectional Encoder Representations from Transformers; TF-IDF: term frequency-inverse document frequency.

The complexity of cases ranges from 1 to 4 with discrete values; thus, we can treat it as either a multi-class classification problem or as a regression problem. The tested models are presented in Figure 6.

For both classification and regression, we used different feature combinations as inputs to train the models. The combinations were as follows: (1) metadata only, (2) word embeddings only, (3) TF-IDF vectors only, and (4) TF-IDF concatenated with metadata.

**Figure 7:**
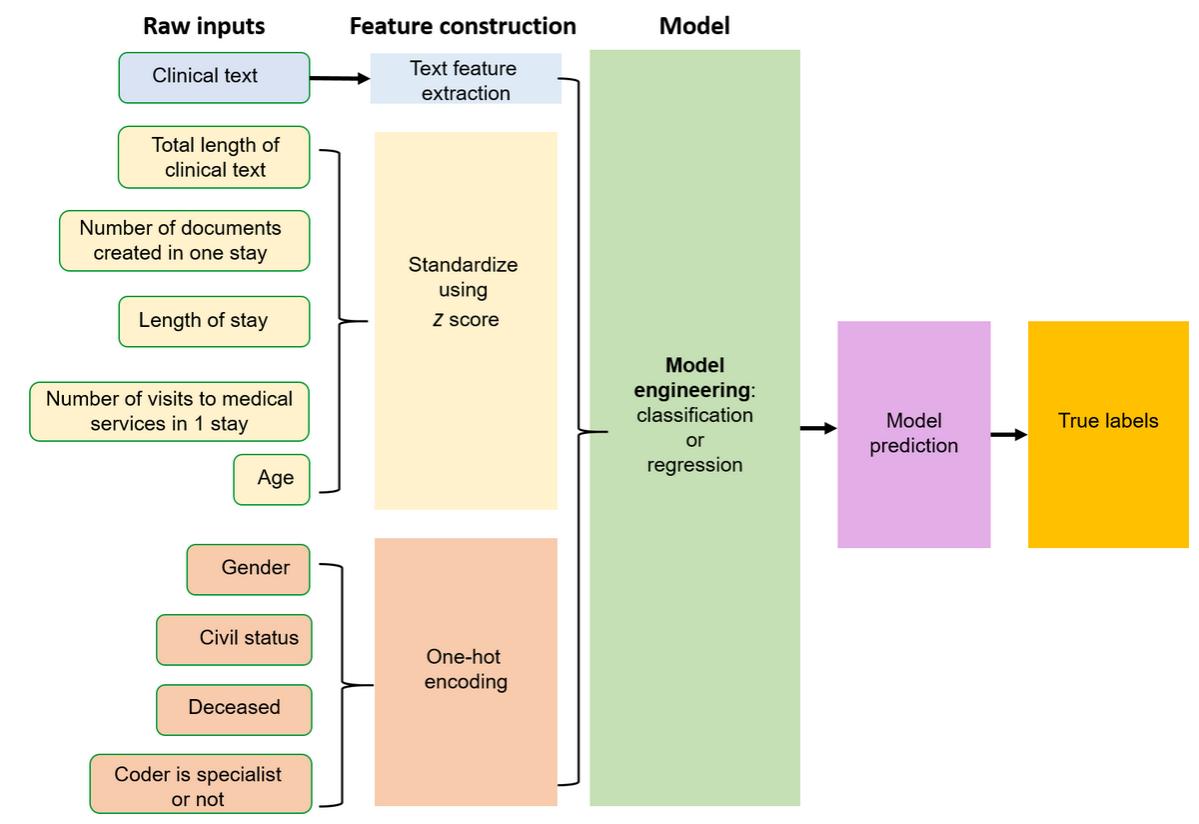
Feature engineering and modeling approach using word embeddings and patient metadata as model inputs. The fine-tuned French-Language Understanding via Bidirectional Encoder Representations from Transformers text classification model is not included in this flow.

The overall process of model implementation is summarized in Figure 7. During training, we applied 5-fold cross-validation to reduce overfitting. As the labels were unbalanced, we used stratified sampling for cross-validation in the classification models. We performed hyperparameter tuning of the most promising features and models. For TF-IDF, we optimized the number of words considered in the vocabulary (topmost frequent words) and text preprocessing (lower case, lemmatization, removal of stop words, and removal of nonalphanumeric tokens). For the gradient-boosted trees model, we tuned the number of estimators, learning rate, and maximum depth. Hyperparameters were tuned based on the average performance over all folds in the cross-validation sets using Bayesian optimization.

In addition, we tested the fine-tuning of the FlauBERT sequence classification model using the Hugging Face transformer library [26]. The FlaubertForSequenceClassification application programming interface provides a pretrained FlauBERT model with a classification layer of size 1024 on top. It takes raw text as input and outputs the predicted classes (in our case, which is the complexity level). Among all our experiments, our best results were obtained using the fine-tuned FlauBERT-base uncased model. Notably, we froze the first 11 encoder layers and trained the last encoder layer and the classification layer to limit overfitting. We also weighted each class differently in the cross-entropy loss to account for imbalance. We used the maximum sequence length of 512 tokens and a batch size of 32. In this manuscript, we only report the fine-tuned FlauBERT results obtained using this configuration.

#### Data Imbalance

Our data labels were strongly imbalanced, and we tried to overcome this issue by using oversampling and undersampling techniques. Our best model was trained using Synthetic Minority Oversampling Technique [27] for oversampling underrepresented classes followed by random undersampling for overrepresented classes. We also chose metrics to penalize models that did not predict underrepresented classes, such as the macro–*F*_1_-score. Ordinal classification can also be an interesting “hybrid” approach. However, we leave trying more sophisticated classification approaches for future work.

#### Technological Stack

The ML pipeline leverages spaCy (version 3.1; Explosion AI) for preprocessing texts (using the French-language model “fr_core_news_md”), scikit-learn (version 1.0.1) to build complex pipelines that can work with cross-validation, and Optuna (version 2.10.0; Preferred Networks, Inc) to conduct hyperparameter searches. It also eases the deployment of the selected model as preprocessing is part of a single serialized pipeline. The other tools used to try other approaches were fastText for document classification, Gensim (RARE Technologies, Ltd) to manipulate pretrained word embeddings, and Hugging Face Transformers (Hugging Face, Inc) to use pretrained transformer models. Training was performed on a virtual machine with 64 central processing unit cores, allowing us to parallelize training, and an Nvidia RTX 3090 graphics processing unit for larger deep learning models.

The first version of the selected model is being deployed with Machine Learning Model Operationalization Management infrastructure in our medical coding service. The deployment details are presented in Multimedia Appendix 1.

## Results

### Metadata Analysis

Each team of coders had a set of medical specialties. We considered that a case was annotated by a specialist if the annotator was part of a team from one of the specialties involved in the case. Following this logic, 63.98% (1318/2060) of the cases were annotated by a specialist. We used this as a feature during training. At inference time, we could choose to request a prediction for whether the case would be coded by a specialist.

The distribution of the numerical metadata and categorical metadata is presented in Figure 5. To check if any of the metadata had significant predictive power on coding complexity, we performed Pearson correlations between the numerical metadata features and the complexity ratings; we also performed statistical tests on categorical features such as patient gender and marital status (Table 1). The results show that, in the precoding phase, features such as sentence length and number of medical services visited during a stay did not have strong effects on coding complexity. In the postcoding phase, the number of ICD-10 codes and Swiss Classification of Surgical Procedures codes showed correlations with coding complexity. With these results, we propose that a future direction of NLP- or AI-assisted coding could use the metadata and clinical text to predict the number of codes that a case may produce and then compare it with the actual codes obtained after the coding process to perform quality checks in the postcoding phase.

**Table 1:** Pearson correlations between the numerical metadata features and the complexity ratings in both the pre- and postcoding phases and statistical tests of the categorical features and complexity ratings in the precoding phase.

			Correlation or statistical test		*P* value
**Numerical features**					
	Number of tokens from all documents in a stay	0.44		<.001	
	Number of documents produced in a stay	0.33		<.001	
	Number of medical services visited during a stay	0.02		.35	
	Duration of the stay	0.41		<.001	
	Age	0.25		<.001	
	Sentence length	0.003		.83	
**Categorical features**					
	Marital status	*F*_5, 2054_=14.05		<.001	
	Gender	t_2058_=−3.70		<.001	
**Other metadata available after coding**					
	Number of ICD-10^a^ codes	0.55		<.001	
	Number of CHOP^b^ codes	0.46		<.001	
	DRG^c^ cost	0.34		<.001	

^a^ICD-10: International Statistical Classification of Diseases and Related Health Problems, 10th Revision.

^b^CHOP: Swiss Classification of Surgical Procedures.

^c^DRG: diagnosis-related group.

### Coder Rating Analysis

**Figure 8:**
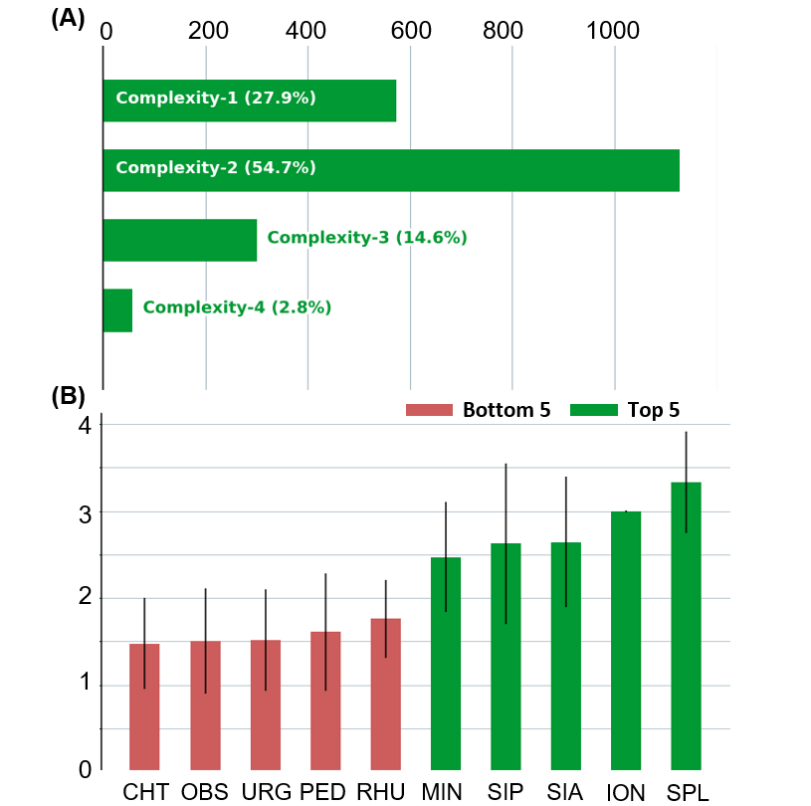
(A) The distribution of complexity ratings over all 2060 cases. (B) Average complexity rating by service. The green bars show the top 5 services, and the red bars show the bottom 5 services. CHT: thoracic surgery; ION: immuno-oncology; MIN: infectious diseases; OBS: obstetrics; PED: pediatrics; RHU: rheumatology; SIA: adult intensive care; SIP: pediatric intensive care; SPL: palliative care; URG: emergency department.

The complexity ratings of the cases are shown in Figure 8A. The most common rating was complexity 2 (1127/2060, 54.71% of cases), and the least common rating was complexity 4 (58/2060, 2.82% of cases). We used stratified sampling to select the training and test sets; hence, their distributions were nearly identical to the true distribution shown in Figure 8A.

The original medical service of a case may also affect its complexity. Figure 8B shows that the cases from the Department of Palliative Care have the highest average complexity, whereas cases from the Department of Thoracic Surgery have the lowest average complexity.

**Figure 9:**
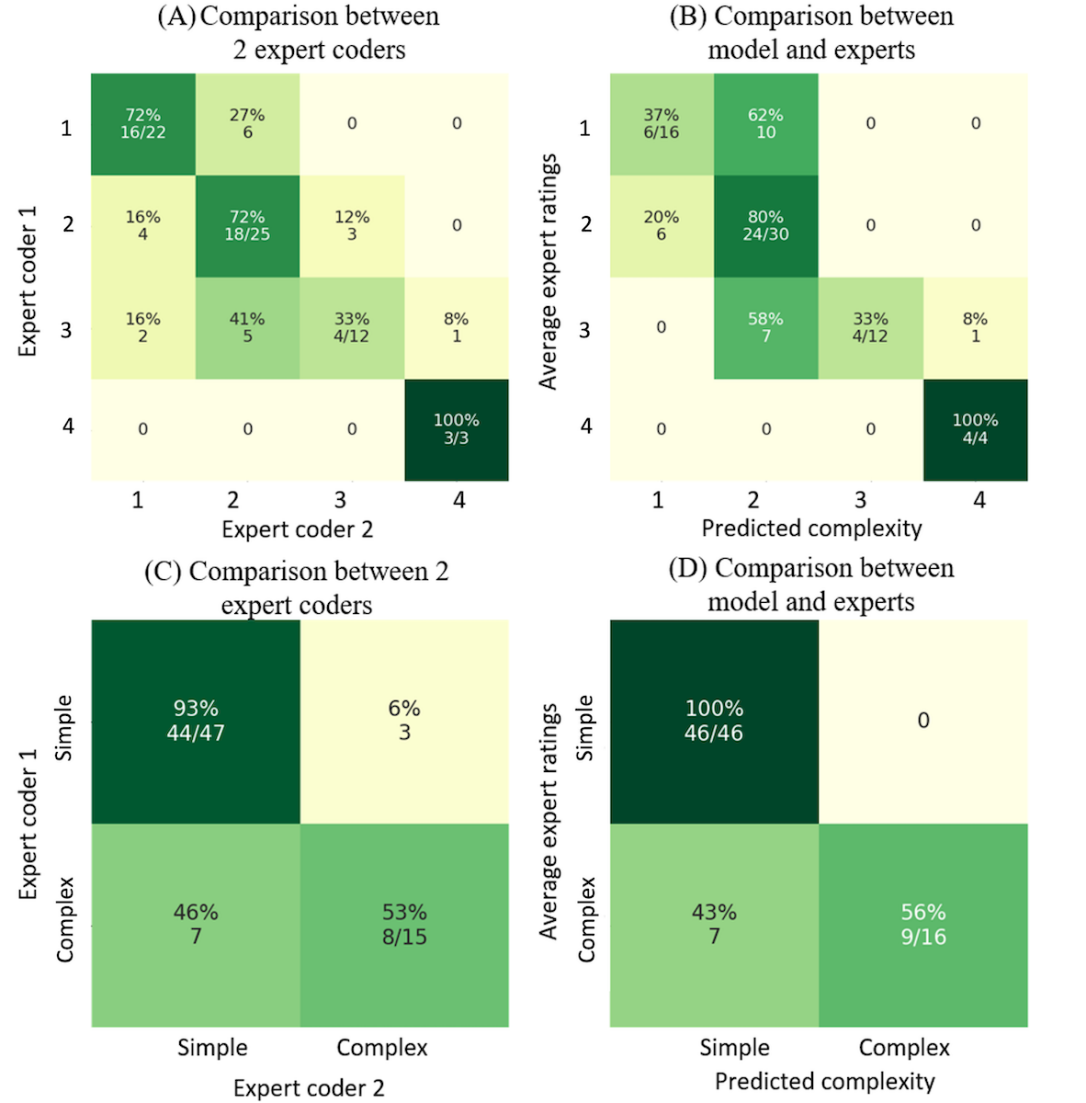
(A) The complexity rating comparison between 2 expert coders on the gold-standard set. (B) The comparison between the validation model’s predictions and average expert ratings on the gold-standard set. (C) The comparison between 2 expert coders’ ratings on the gold-standard set when grouping into simple (complexity 1 and 2) and complex (complexity 3 and 4) cases. (D) The comparison between average expert ratings and the validation model’s predictions on the gold-standard set when grouping into simple and complex cases. The average expert ratings are rounded up to the next largest integer.

By analyzing the gold-standard set, where all cases were rated by 2 experts, we found that even the expert coders did not always agree with each other. Of the 62 cases, the 2 experts agreed on 41 (66%). However, they disagreed by more than one complexity level in only 3% (2/62) of cases (Table 2). The interrater reliability (Cohen κ score) was 0.49 between the 2 expert coders. If we consider one expert as the ground truth and the other expert as a predictive model, the macro–*F*_1_-score of this “predictive model” can only achieve 0.67 (Figure 9), a moderately good score showing that the task can be learned but models will not achieve a very high performance.

The reason why coders rate the same case with different complexity levels is mainly subjectivity. This is also a reminder that subjective-rated labels are often noisy, and no model can achieve a perfect performance. The ratio of agreement between 2 expert coders gives us an idea of the performance we could expect from a model. If we consider one expert as the model that predicts complexities and the other expert gives true complexity labels, then the highest accuracy that this model (the former expert) can achieve is 66%. In this sense, when later analyzing our model’s performance, the 66% accuracy can be considered as one of the benchmarks. However, given the strong imbalance in the complexity labels, we should rely as well on the confusion matrix to compare the annotator-annotator agreement with the model-annotator agreement.

However, as mentioned in the Model Design section, our samples were highly imbalanced, and the accuracy metric lacked the ability to measure the model’s performance comprehensively according to the sample distribution. As there were 54.71% (1127/2060) of cases rated with a complexity of 2, a naive model that predicts 2 all the time could reach an accuracy of 54.71%, but it provides no value for solving our problem. To consider the imbalanced sample distribution, we used the macro–*F*_1_-score together with accuracy to measure the model performance. The macro–*F*_1_-score between the 2 coders was 0.67, which was considered as the other benchmark that we used to evaluate the model’s performance.

**Table 2:** Absolute difference between expert 1 and expert 2 complexity ratings. The accuracy reached by expert coders was approximately 66% (41/62; N=62).

Absolute difference in complexity ratings between expert coders 1 and 2 (number of complexity levels)	Cases, n (%)
0	41 (66)
1	19 (31)
2	2 (3)
3	0 (0)

### Model Analysis

#### Overview

First, we wanted to study whether our approach worked on predicting coding complexity for medical cases. We made use of all the 2060 annotated cases (n=1998, 96.99% 1-coder–rated and n=62, 3.01% gold-standard cases). We split the 2060 cases into a training set (n=1751, 85% of cases) and a test set (n=309, 15% of cases) and tested our model architecture. Then, to validate the model’s performance with expert coders’ benchmarks, we left the 3.01% (62/2060) of gold-standard cases out as the test set and trained a model with the same architecture but with more training data (1998/2060, 96.99% of cases).

#### The Main Model

To train the models, we started by using either patient metadata only or word embeddings or TF-IDF vectors only as input features. The best-performing model using patient metadata was gradient-boosted trees (macro−*F*_1_-score=0.46; accuracy=0.61 for classification; *R*^2^=0.15 for regression). The best-performing model using word embeddings was the fastText classification model (macro−*F*_1_-score=0.47; accuracy=0.57; initialized with customized embeddings), and the best-performing model using TF-IDF vectors was gradient-boosted trees (macro−*F*_1_-score=0.45; accuracy=0.62 for classification; *R*^2^=0.26 for regression).

The model using word embeddings did not outperform the model using TF-IDF vectors. Thus, we combined the TF-IDF vectors with metadata as input features to integrate information from both patient metadata and medical dossiers. The best-performing model used gradient-boosted trees and achieved a macro−*F*_1_-score of 0.51 and accuracy of 0.59 on the cross-validated training set and a macro−*F*_1_-score of 0.46 and accuracy of 0.58 on the test set. Figure 6 shows the performance comparison between different models on the 5-fold–cross-validated training data set and the test set. The detailed numbers can be found in Multimedia Appendix 1.

As performing well on underrepresented classes is important in our case, we report the macro–*F*_1_-score as the first metric. Macro–*F*_1_-score is the average of the *F*_1_-score per class and is not weighted by the number of instances in the class. Unlike accuracy, this metric penalizes each class equally. On the basis of the macro–*F*_1_-score, we selected our best model as the gradient-boosted trees trained with the combined TF-IDF and metadata features (referred to as the main model).

**Figure 10:**
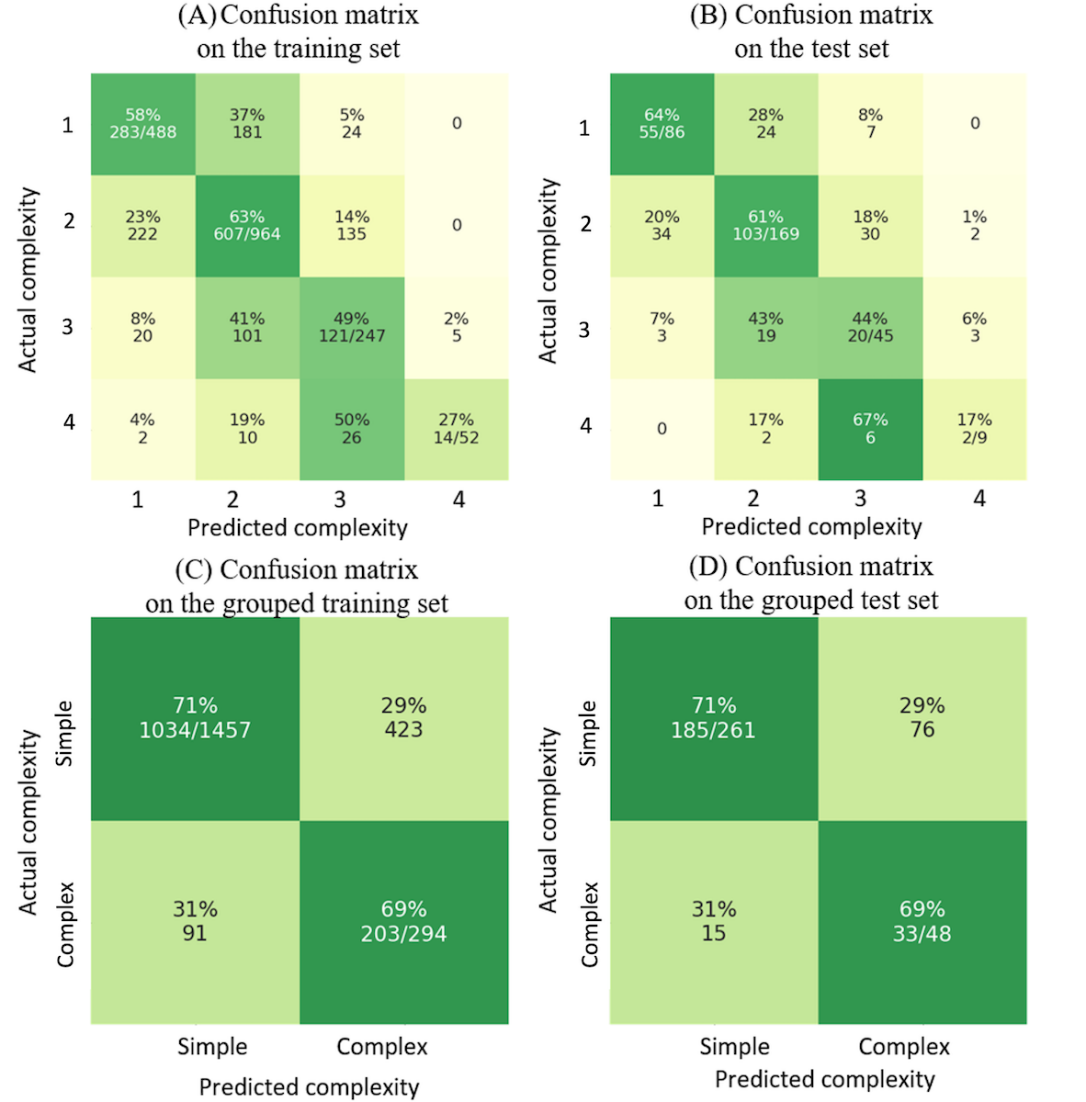
(A) and (B) The main model’s performance on the training set (1751 cases) and the test set (309 cases). (C) and (D) The main model’s performance on the grouped training set (1457 cases as simple and 294 cases as complex) and the test set (261 cases as simple and 48 cases as complex).

The confusion matrix (Figures 10A and 10B) shows that our main model confused complexity-2 and complexity-3 cases during training and testing. Figure 9A shows that, even for expert coders, there was no clear distinction when rating complexity 2 and 3 for a case. The difficulty to distinguish between complexity 2 and 3 could be due to the similarity between the 2 classes of cases. We noticed that our main model also had difficulties distinguishing between complexity 3 and 4 during training and testing. This performance could be due to the lack of examples. Although we performed oversampling using Synthetic Minority Oversampling Technique on cases with a complexity of 3 and 4, it still lacked variability in complexity-4 cases.

We then tried to merge complexity-1 and complexity-2 cases as “simple” cases and complexity-3 and complexity-4 cases as “complex” cases and tested the model as a binary classifier. The results (Figures 10C and 10D) show that the model performed well on distinguishing between simple and complex cases. On the training set, the model achieved a macro–*F*_1_-score of 0.62 with an accuracy of 0.71. On the test set, the model achieved a macro–*F*_1_-score of 0.65 with an accuracy of 0.71.

#### The Validation Model

To validate our model approach and compare it with experts’ benchmarks, we trained a validation model using the 96.99% (1998/2060) of 1-coder–rated cases and tested it on the 3.01% (62/2060) of gold-standard cases. The architecture of the validation model was the same as that of the main model.

The comparison between the 2 expert coders’ ratings (Figure 9A) shows that most of the expert coders’ disagreements were on complexity-2 and complexity-3 cases, and the overall agreement ratio between the 2 coders was 66% (41/62), with a macro–*F*_1_-score of 0.67. Table 3 and Figure 9B show the comparison between our validation model and the 2 experts’ ratings on the gold-standard set. The model agreed on 53% (33/62) of the cases with expert coder 1 and in 63% (39/62) of the cases with expert coder 2. The validation model achieved a 61% agreement ratio with the average ratings of both experts, with a macro–*F*_1_-score of 0.62.

**Table 3:** Comparison between our validation model’s predictions and 2 expert coders’ ratings on the gold-standard set.

	Percentage of agreement	Pearson correlation
Expert coder 1 vs expert coder 2	66	0.70^a^
Model vs expert coder 1	53	N/A^b^
Model vs expert coder 2	63	N/A
Model vs ceiled mean of 2 expert coders	61	0.70^a^

^a^*P*<.001.

^b^N/A: not applicable.

When merging the 4 complexity levels into 2 (simple vs complex; Figures 10C and 10D), the agreement ratio between the 2 coders became 84% (52/62) with a macro–*F*_1_-score of 0.76, and the agreement ratio between model predictions and average expert ratings became 0.89 with a macro–*F*_1_-score of 0.82. The results indicate that the model is comparable with human experts’ performance and predicts in a very similar manner to that of human experts (Figures 9A and 9B).

Interestingly, for the gold-standard cases, our validation model managed to predict complexity-4 cases 100% correctly, which was different from the main model’s performance during training and testing (Figures 10A and 10B). As there were only 4 selected cases with a complexity of 4 owing to the sampling for expert cases, these cases could be extremely complex and, thus, easy for the model to identify.

Compared with other models that can provide higher accuracy but lower *F*_1_-score, both the main model and the validation model were more practical in our concrete use case as it is important to predict diverse complexity levels rather than keep predicting a complexity of 2 for all cases (Multimedia Appendix 1).

#### Classification Versus Regression

We summarize the pros and cons of both approaches given our use case in Textbox 4.

**Pros and cons of the classification and regression approaches.**

**Figure 11:**
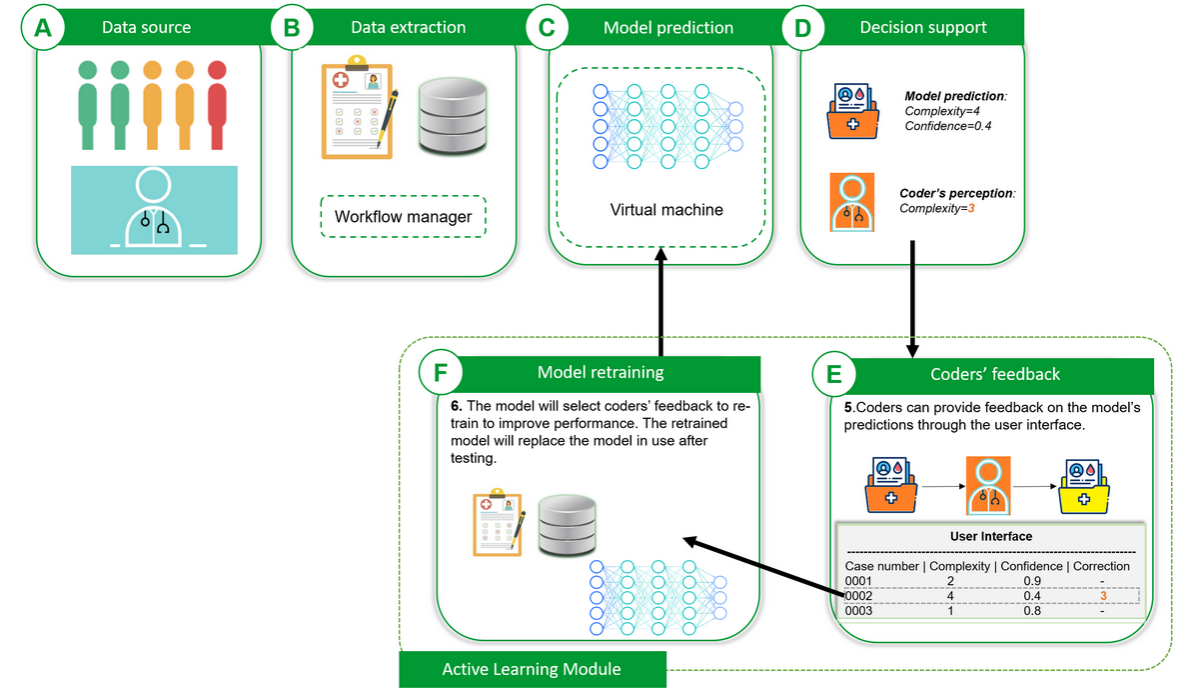
Use of active learning module to collect coders’ feedback and improve model performance. The workflow manager in (B) can be any software or platform that provides automatic scheduling for designated work (eg, a script for data extraction).

- *Prediction confidence*: many classification models output the confidence in the predicted class as a probability, whereas regression models typically do not provide such information out of the box (although CIs are sometimes possible). Confidence is useful for end users, meaning that they can disregard predictions with low confidence. It can also be used in the active learning module (Figure 11) to select new cases (with low prediction confidence and strong disagreement between prediction and coder perception) to retrain the model.
- *Interpretability of results*: using a classification approach enables the computation of *F_1_*-scores, accuracy, and confusion matrices. These are more intuitive for end users. Note that, for regression, it is still possible to round prediction to apply these metrics.
- *Order of labels*: complexity scores are naturally ordered. Therefore, given a case annotated with a complexity of 4, a model should be penalized more for predicting a complexity of 1 than for predicting a complexity of 3. Regression methods consider order, whereas classification methods do not.

## Discussion

### Principal Findings

We presented different ML models that can predict the complexity of coding medical cases with 4 complexity levels. We first trained the models on all 2060 annotated cases. When only using patient metadata, the best model (gradient-boosted trees) could achieve a macro−*F*_1_-score of 0.46, an accuracy of 0.61 for classification, and an *R*^2^ of 0.15 for regression. By applying NLP methods to extract information from clinical text, the best model (fastText initialized with customized embeddings) could achieve a macro−*F*_1_-score of 0.47 and an accuracy of 0.57 for classification. When combining patient metadata and NLP-extracted information, the best model (the main model in the Model Analysis section) achieved a macro−*F*_1_-score of 0.51 and an accuracy of 0.59 on the cross-validated training set and a macro−*F*_1_-score of 0.46 and an accuracy of 0.58 on the test set.

To evaluate our model approach with experts’ benchmarks, we trained our validation model using the same architecture as the main model on all except the gold-standard cases. Our validation model achieved an accuracy of 0.61 with a macro−*F*_1_-score of 0.62 on the gold-standard cases. When merging the 4 complexity levels into “simple” (complexity 1-2) and “complex” (complexity 3-4) cases, our validation model could achieve an accuracy of 0.89 and a macro−*F*_1_-score of 0.82. The results indicate that the model performance is highly comparable with that of human experts.

To the best of our knowledge, this is the first study to apply NLP and ML models to help differentiate the complexity of coding medical cases.

### Clinical Importance

Lausanne University Hospital in Switzerland has 2 missions: guaranteeing medical services in an area and serving as a referral hospital. The dominance of cases with a complexity level of 2 (referred to as case 2) in the labeled sample cases can be explained by this double activity as the hospital not only concentrates on university or referred complex cases but also receives normal cases similar to other hospitals.

In our current medical coding service, the cases to be coded are distributed 50% to the team of the specialty and 50% to a “common pot.” This team versus common pot distribution is done randomly without considering the complexity of the cases, leaving complex cases in the common pot and, conversely, depriving the common pot of “simple” cases of specialized resources. Note that, in our case, coders can still choose complex cases from the common pot even if the case is not in their specialty. Many coders care about diversity or learning other types of cases. The integration of this model enables them to choose the complexity consciously.

The dominance of cases 2 will have the effect of pushing a lot of cases into the common pot, reducing the number of cases arriving to teams of different specialties and, hence, reducing the ratio of common pot to specialists. The quality of coding of complexity-3 and complexity-4 cases will be improved as they will be redirected to the specialty teams or senior coders. However, this will also be at the risk of lowering the quality of coding of cases 2, which will end up in the common pot. Therefore, it will be necessary to maintain a 50/50 ratio between the common pot and the teams or senior coders and force cases 2 to be coded by teams or seniors as well. This adjustment will enhance the quality of coding of cases 3 and 4 and a maximum of cases 2. After our system is deployed, the new distribution considering the complexity predicted by our NLP and ML model will be monitored in terms of satisfaction of the coding teams and accuracy of coding. Furthermore, we will analyze the accuracy of coding in relation to the predicted case complexity to adjust the model design and more efficiently allocate the case distribution to coders.

In our current model, the complexity of the cases is defined by the coders from our medical service and is rated subjectively. By analyzing the model predictions for a variety of cases, it is possible to summarize the common features shared by the high-complexity cases and those shared by the low-complexity cases. The summarized features can be used to build a set of objective rules that can be shared with other clinical services or the medical coding services of other hospitals. For small hospitals or clinical services, which do not always have sufficient resources to train and build their own ML models, this set of rules can help them distribute the cases more efficiently. In contrast, if the summarized features could not distinguish well between the simple and complex cases, it may reflect that the case complexity is a subjective rather than objective measure. In this situation, the best way to generalize this subjective measure is to build a model, such as in our approach, to learn the highly nonlinear subjective measures.

The complexity of coding a medical case can approximately reflect the complexity of the corresponding clinical case. Our application can not only improve resource allocation in medical coding services but also be generalized to other clinical services. Indeed, coding complexity levels can also be used in decision-making processes to help arbitrate resource allocation among professionals in the same department but affiliated with different clinical services within the department. For example, in the surgery department, a similar approach can be applied to help study the need for resources for different subspecialties based on the volume of treated cases but also on their relative complexity. The generalized application can be integrated into different digital health care systems for automatic task assignment to avoid conflicts in an unfair workload distribution.

### Technical Importance

OOV is an issue that can impair model performance. Although the word2vec embeddings used in this study were trained on our own clinical data, OOV was still present as the corpus we used to train the embeddings might not have been sufficient to cover all the clinical terms used in the medical discharge documentation. To mitigate the impact of OOV, we tested the fastText subword approach. However, as shown in the Model Analysis section, the model performance was not much improved because of the low OOV ratio of our data set, which was only approximately 8% in the 2060 selected cases for this study. We provide a detailed analysis of OOV in our corpus in Multimedia Appendix 1.

As new clinical documents are produced every day, our deployed model could also face the impaired performance caused by the OOV issue. The solution we propose in this paper to reduce the impact is to monitor the evolution of new OOV with respect to the training data set and retrain the word embeddings when needed. During the retraining phase, we will not only retrain the word embeddings but also retrain the models with coder feedback to further improve the model performance from the perspective of both feature engineering and model engineering.

In our study, we used FlauBERT, which is a pretrained French-language transformer, in 2 different ways. The first way to use it is to generate word embeddings as text features for model inputs. We then also tested a Hugging Face [26] implementation of the sequence classification model using FlauBERT. A detailed description of this approach is presented in Multimedia Appendix 1. The best performance using the transformer model directly achieved a macro–*F*_1_-score of 0.47, which is similar to other models that only receive text as features. The model performance did not improve as much as expected. The reason could be that our data set was too small (only 2060 cases) compared with the size of the transformer model. Regarding this, we will continue collecting coder feedback on the predicted cases and use them to train the model continuously. With these approaches, we hope to improve the transformer model performance in the future.

We found that using TF-IDF vectors as text features provided better prediction performance than using word embeddings as text features. The fastText and FlauBERT embeddings were pretrained on a nonclinical corpus; thus, the represented context of the word could deviate from the context used in the clinical text. As shown in the Metadata Analysis section, the median document length per stay was 909 tokens. Common pretrained transformer-based models handle up to 512 tokens, and it is not obvious which subset of the document should be selected to pass to the model. Although it is possible to overcome this limitation by embedding each chunk of 512 tokens and averaging their embeddings, we believe that a substantial improvement over other methods is needed to justify the computation cost. Furthermore, fastText and word embeddings both perform averaging over all vectors of each document, which may dilute the signal too much given the number of tokens. In contrast, TF-IDF can preserve some of this information, which could be the reason why TF-IDF vectors outperformed word embeddings in our task. A future direction to improve the model performance could be to combine TF-IDF vectors with word embeddings as text features. TF-IDF vectors can be used as a weight of importance for the words, whereas word embeddings can represent the contexts of the words. By combining the two, we could obtain vectors that represent both the importance and context of the words comprehensively. Another possible approach to improve the model performance is to build a rule-based model from coders’ experiences and then combine the rule-based model with the ML model, which can increase both the interpretability and flexibility of the prediction. As the complex cases are more likely to have multiple laboratory tests and clinical examinations, we could also include this structured clinical information for future feature engineering.

By comparing our model’s predictions with the expert coders’ ratings, we found that the model could achieve an expert performance level (Figure 9). As rating case complexity is relatively subjective, even expert coders do not always agree with each other. This introduced another level of complexity to our study. However, by learning 1998 cases from the training set, our model’s performance became comparable with that of the experts.

One of the advantages of our model is that we used a multimodal approach. Structured data such as patient metadata can provide quantitative information about patients’ status. Clinical text can provide rich information on diagnostic and other assessments of patients, which are not usually presented in the structured data. By combining the two, we are able to maximize the information needed to evaluate the complexity of a clinical case. Our study used 1 model to process data of different modalities and make predictions. In future work, we propose using dedicated models for each data modality and combining the predictions of multiple models using another ML model to make the final prediction. The benefits of using multiple models are that (1) it is easy to plug in new data and new models into the architecture, which makes the model flexible to extend, and (2) it is easier to perform feature engineering and interpret the model’s prediction.

The advantage of classification models over regression models in our study was that classification models allowed us to produce the confidence of the predictions. By showing both the predicted complexity level and the confidence of the prediction, we are able to provide comprehensive information to end users. However, there are also limitations to our model. Of the 2060 cases we collected for this project, 54.71% (1127/2060) were labeled as complexity-2, and only 2.82% (58/2060) were labeled as complexity-4. The unbalanced data set affects the performance of the classification models, meaning that the models have a higher tendency to predict complexity 2 for a given case. This problem was tackled by oversampling the underrepresented cases and undersampling the overrepresented cases. The results showed that the model performed better with oversampling and undersampling techniques (Multimedia Appendix 1).

Our model will be integrated into our current coding system with an active learning module. Figure 11 shows the integration architecture. The model reads patient metadata and medical dossiers regularly from our clinical data warehouse through a workflow manager. The predictions are presented in the user interface of the coding software. When coders find that the prediction deviates from the perceived complexity, they can put their corrections in a feedback field. Coders’ feedback is stored and sent to the model for retraining. This integration architecture allows us to track and continuously improve the performance of the model.

### Future Work

Future work can be carried out on different aspects. To improve the model prediction performance, we can continue working on feature and model engineering. In addition to the data we used in this study, there could be other patient data that can be useful to predict the complexity of cases. Regarding the text features, we could try different combinations of NLP tools to maximize the information extraction from clinical text. We will also continue working on reducing the OOV impact by retraining the word embeddings (both word2vec and fastText) and TF-IDF vectors every 6 months and use coder feedback as new training samples to retrain the models. To make full use of the advanced transformer models, we will not only keep training using the new samples but also explore ways to incorporate patient metadata into the model design. We will also work together with coders to establish a sound and interpretable rule-based model and then combine it with the ML model. The hybrid model can provide both flexibility and good reasoning in distinguishing cases.

Currently, most NLP applications focus on AI-assisted coding using rule-based or ML models. As stated before, the rules framing medical coding complexity are dynamic and change over time, preventing the rapid learning of the tool. Instead of using AI-assisted tools only for coding, it is possible to extend the AI-assisted scope from case preselection to postcoding quality checks. Our approach provides a possibility to preselect cases that are suitable for automatic coding and other cases for manual coding. After a case is coded, AI-assisted tools can provide a post hoc analysis of the code categories and combinations, aiming to find possible mistakes in the codes. This can be done by studying previous coded cases using statistical and NLP analysis.

We also aim to continuously evaluate the application’s impact on our medical coding service. After the integration, we will monitor the average time a coder spends coding a case and the average number of mistakes a coder makes for each case. By comparing the time and accuracy before and after the integration, we can obtain a quantitative measure of how much improvement the model can bring to the coders’ daily work.

In addition to monitoring the quality of coding, we will keep tracking the coders’ user experience. With the help of the active learning module, we are able to collect coders’ feedback on the model’s predictions. The model will be retrained based on coders’ feedback through iterations to improve the prediction performance. As discussed in the Clinical Importance section, our application can not only help with task distribution to current coders but also be used to select cases for training junior coders. Junior coders will receive simple cases at the beginning and gradually receive more complex cases. This approach can give junior coders enough exposure to a variety of cases with respect to their capabilities as well as evoke their interests in medical coding.

## Appendix

Multimedia Appendix 1 Illustrations on the text feature engineering, imbalanced data processing, MLOps infrastructure, model comparison table, OOV analysis, and transformers fine-tune methods.

## Abbreviations

AI: artificial intelligence
FlauBERT: French-Language Understanding via Bidirectional Encoder Representations from Transformers
ICD-10: International Statistical Classification of Diseases and Related Health Problems, 10th Revision
ML: machine learning
NLP: natural language processing
OOV: out of vocabulary
TF-IDF: term frequency-inverse document frequency
