# Correction: An End-to-End Natural Language Processing Application for Prediction of Medical Case Coding Complexity: Algorithm Development and Validation

**DOI:** 10.2196/94047

**Published:** 2026-04-14

**Authors:** He Ayu Xu, Bernard Maccari, Hervé Guillain, Julien Herzen, Fabio Agri, Jean Louis Raisaro

**Affiliations:** 1 Biomedical Data Science Center Lausanne University Hospital Lausanne Switzerland; 2 Unit8 SA Lausanne Switzerland; 3 Public Health Solutions Ltd Promasens Switzerland; 4 Department of Administration and Finance Lausanne University Hospital Lausanne Switzerland; 5 Department of Visceral Surgery Lausanne University Hospital Lausanne Switzerland

In “An End-to-End Natural Language Processing Application for Prediction of Medical Case Coding Complexity: Algorithm Development and Validation” [1] the authors made two corrections. The y-axis labels in Figures 9 and 10 were misprinted and did not correspond to the intended values shown in the original submission. The underlying data and conclusions of the paper remained unchanged.

The first figure originally appeared as follows (Figure 1):

**Figure 1:**
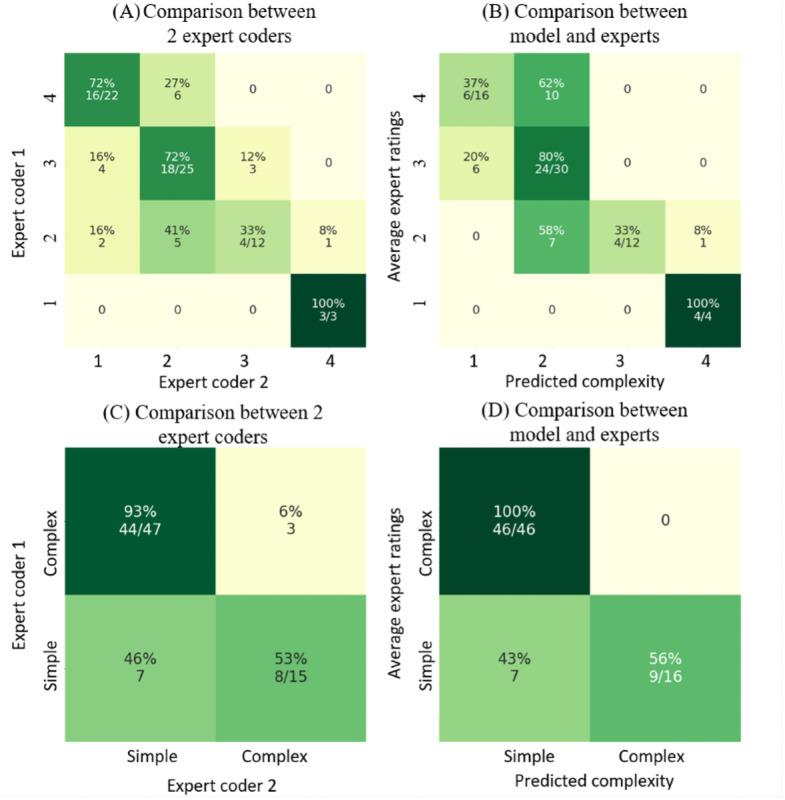
Original Figure 9, (A) The complexity rating comparison between 2 expert coders on the gold-standard set. (B) The comparison between the validation model’s predictions and average expert ratings on the gold-standard set. (C) The comparison between 2 expert coders’ ratings on the gold-standard set when grouping into simple (complexity 1 and 2) and complex (complexity 3 and 4) cases. (D) The comparison between average expert ratings and the validation model’s predictions on the gold-standard set when grouping into simple and complex cases. The average expert ratings are rounded up to the next largest integer.

This figure has been revised to the following (Figure 2):

**Figure 2:**
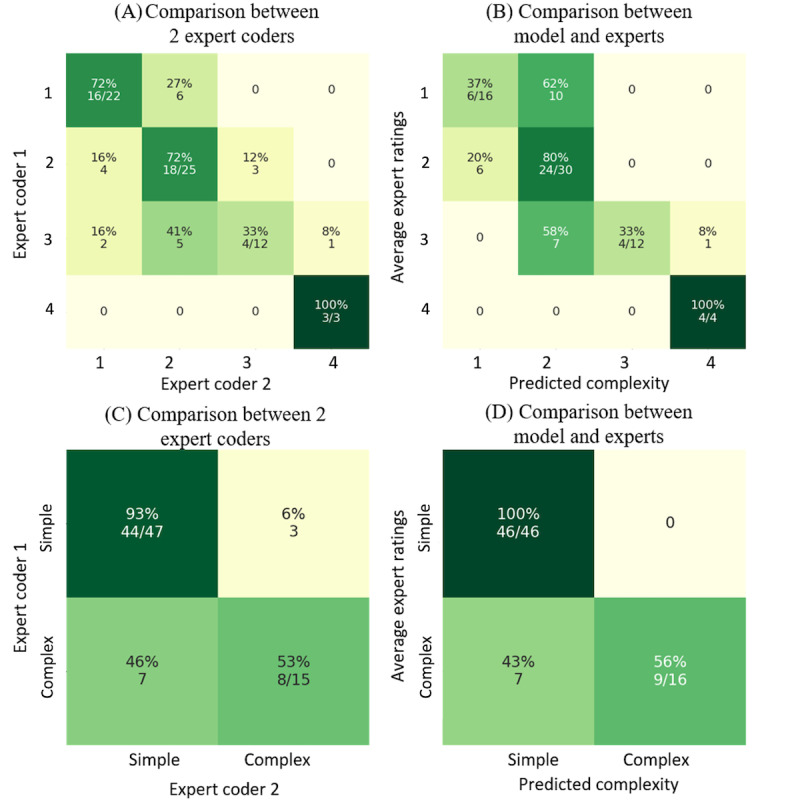
Revised Figure 9, (A) The complexity rating comparison between 2 expert coders on the gold-standard set. (B) The comparison between the validation model’s predictions and average expert ratings on the gold-standard set. (C) The comparison between 2 expert coders’ ratings on the gold-standard set when grouping into simple (complexity 1 and 2) and complex (complexity 3 and 4) cases. (D) The comparison between average expert ratings and the validation model’s predictions on the gold-standard set when grouping into simple and complex cases. The average expert ratings are rounded up to the next largest integer.

The y-axis labels in Figure 9 (A, B) were presented in reverse order. In the original published version, the labels appeared as 4, 3, 2, 1 from top to bottom, whereas they should read 1, 2, 3, 4 from top to bottom. The y-axis labels in Figure 9 (C, D) were presented in reverse order. In the original published version, the labels appeared as “Complex, Simple” from top to bottom, whereas they should read “Simple, Complex” from top to bottom. The underlying data and conclusions of the paper remained unchanged; however, the labeling error may cause confusion for readers.

The second figure originally appeared as (Figure 3):

**Figure 3:**
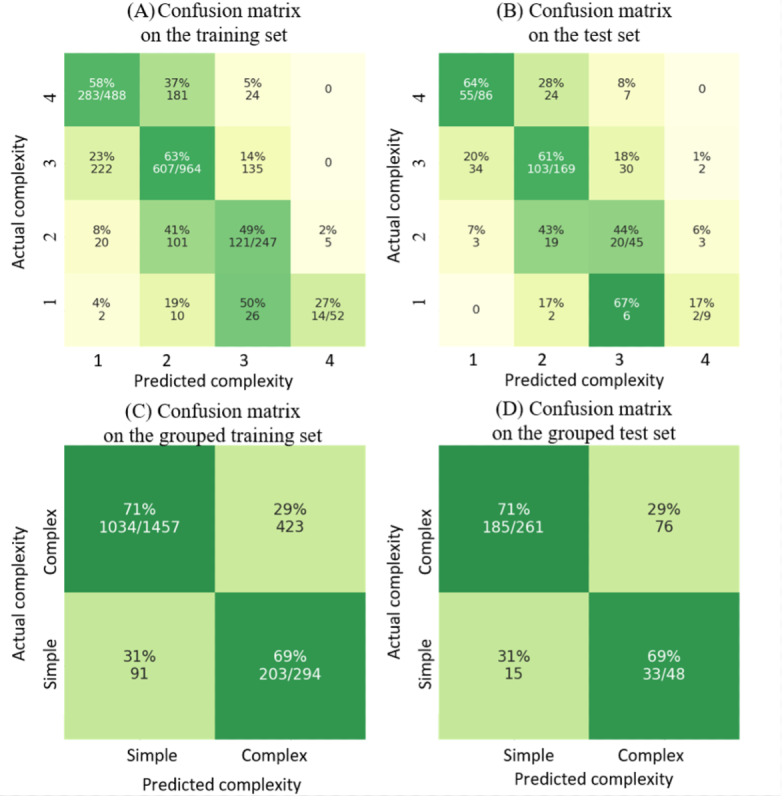
Original Figure 10, (A) and (B) The main model’s performance on the training set (1751 cases) and the test set (309 cases). (C) and (D) The main model’s performance on the grouped training set (1457 cases as simple and 294 cases as complex) and the test set (261 cases as simple and 48 cases as complex).

This figure has been revised to the following (Figure 4):

**Figure 4:**
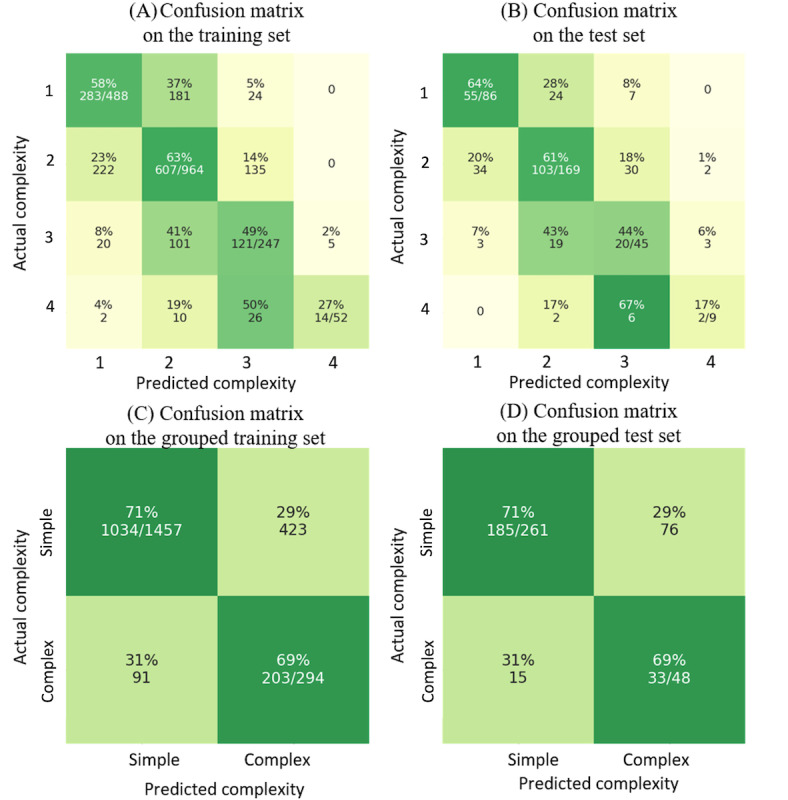
Revised Figure 10, (A) and (B) The main model’s performance on the training set (1751 cases) and the test set (309 cases). (C) and (D) The main model’s performance on the grouped training set (1457 cases as simple and 294 cases as complex) and the test set (261 cases as simple and 48 cases as complex).

The y-axis labels in Figure 10 (A, B) were presented in reverse order. In the published version, the labels appeared as 4, 3, 2, 1 from top to bottom, whereas they should read 1, 2, 3, 4 from top to bottom. The y-axis labels in Figure 10 (C, D) were presented in reverse order. In the published version, the labels appeared as “Complex, Simple” from top to bottom, whereas they should read “Simple, Complex” from top to bottom. The underlying data and conclusions of the paper remained unchanged; however, the labeling error may cause confusion for readers.

The correction will appear in the online version of the paper on the JMIR Publications website, together with the publication of this correction notice. Because this was made after submission to PubMed, PubMed Central, and other full-text repositories, the corrected article has also been resubmitted to thos
