# Supplementary material for: An End-to-End Natural Language Processing Application for Prediction of Medical Case Coding Complexity: Algorithm Development and Validation
Source: JMIR Med Inform. 2023 Jan 19;11:e38150. doi: 10.2196/38150 (PMC9896350; doi:10.2196/38150)
Supplement: Multimedia Appendix 1 [file medinform_v11i1e38150_app1.docx]

Multimedia Appendix (Supplementary Materials)

1. Text Feature Engineering
2. *TF-IDF*

Term Frequncy – Inverse Document Frequency (TF-IDF) measures the importance of a word in a corpus based on the frequency the word appears.

The term frequency (TF) is measured as:

$tf\left( t, d \right)= \frac{f_{t,d}}{\sum_{t'\in d} f_{t'd}}$

Where ***t*** presents the term, ***d*** presents the document, $f_{t'd}$ presents the number of times that term ***t*** appears in document ***d***.

The inverse document frequency (IDF) is measured as:

$idf\left( t, D \right)= log\frac{N}{\left| \left\{ d\in D:t\in d \right\} \right|+1}$

Where ***N*** presents the total number of documents in the corpus, $|\{d\in D:t\in d\}|$ presents the number of documents where term ***t*** appears, to avoid division by zero, it is common to add 1 to $|\{d\in D:t\in d\}|$.

The final TF-IDF is computed as:

$tfidf\left( t, d,D \right)= tf\left( t,d \right)*idf(t,D)$

1. *Transform text to vectors*

The transformation from text to TFIDF vectors is done by using scikit-learn python package. We considered the maximum vocabulary size is 1,000, so the computed TF-IDF vector of each document is of size 10,000.

The customized word2vec embedding is trained using python package gensim. The size of the vector is 100. Each word of a given document is converted to a vector of size 100. The vector that represents the whole document is computed by averaging the vectors of all words in the document.

The python package fastText provides a text classification module that can be used directly to train and predict the labels of given documents. We initialized the fastText module using our pre-trained word2vec embeddings. The vector that represents a document is of size 100.

We used the transformer model FlauBERT from the HuggingFace module. We took the first 512 tokens of each document as the input. Each token is encoded into one vector of size 768, and the vector that represents the whole document is computed by averaging the vectors of all tokens or by taking the CLS (pooled output).

1. Oversampling and undersampling on imbalanced data

*Dealing with imbalance:* To attempt to overcome the strong class imbalance, we tried two common approaches. Firstly, we tried adjusting the weights of samples inversely proportional to the class frequencies in the training set. The exact weighting mechanism depends on the algorithm but intuitively they all attempt to give more weight to inputs of underrepresented classes. This did not yield any noticeable improvement. Secondly, we tried oversampling methods: Random over-sampling which augments the training set with duplicate samples of the underrepresented classes to reach class balance, and SMOTE, which creates synthetic examples from examples of the underrepresented classes. This yielded limited improvements. Thirdly, we tried random undersampling, which yielded the most improvements. Other undersampling methods like Tomek Links did not perform as well. The best approach was to oversample class 3 (3 times) and 4 (4 times) with SMOTE then undersample randomly class 1 (0.8 times) and 2 (0.5 times). Without resampling, we obtain a similar macro-f1 but the model tends to always predict 2 and confuses 2 and 3 which significantly reduces the value for the end user.


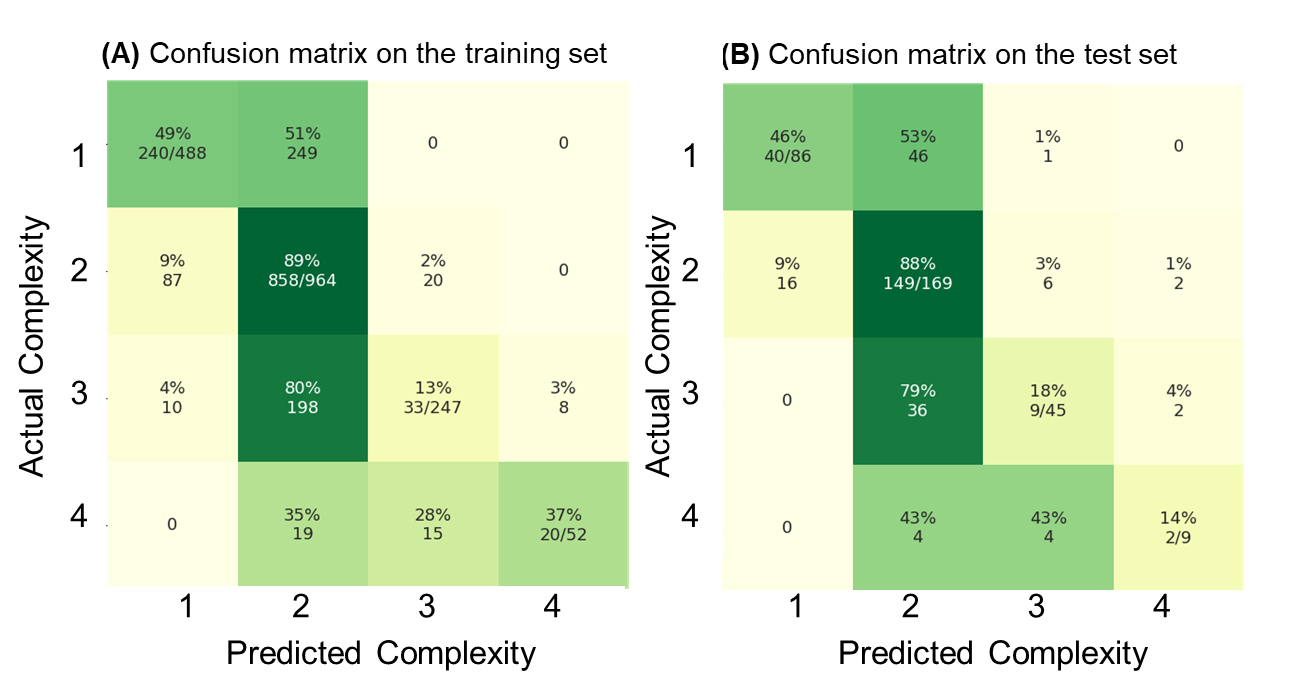


Figure S12 Model performance on the training set and the test set, trained without oversampling or undersampling strategies.

*Other algorithms*: We tried training a feed-forward neural network, a random-forest, a support vector machine both for classification and regression. They all performed slightly worse than the Gradient boosted trees approach.

1. MLOps Infrastructure


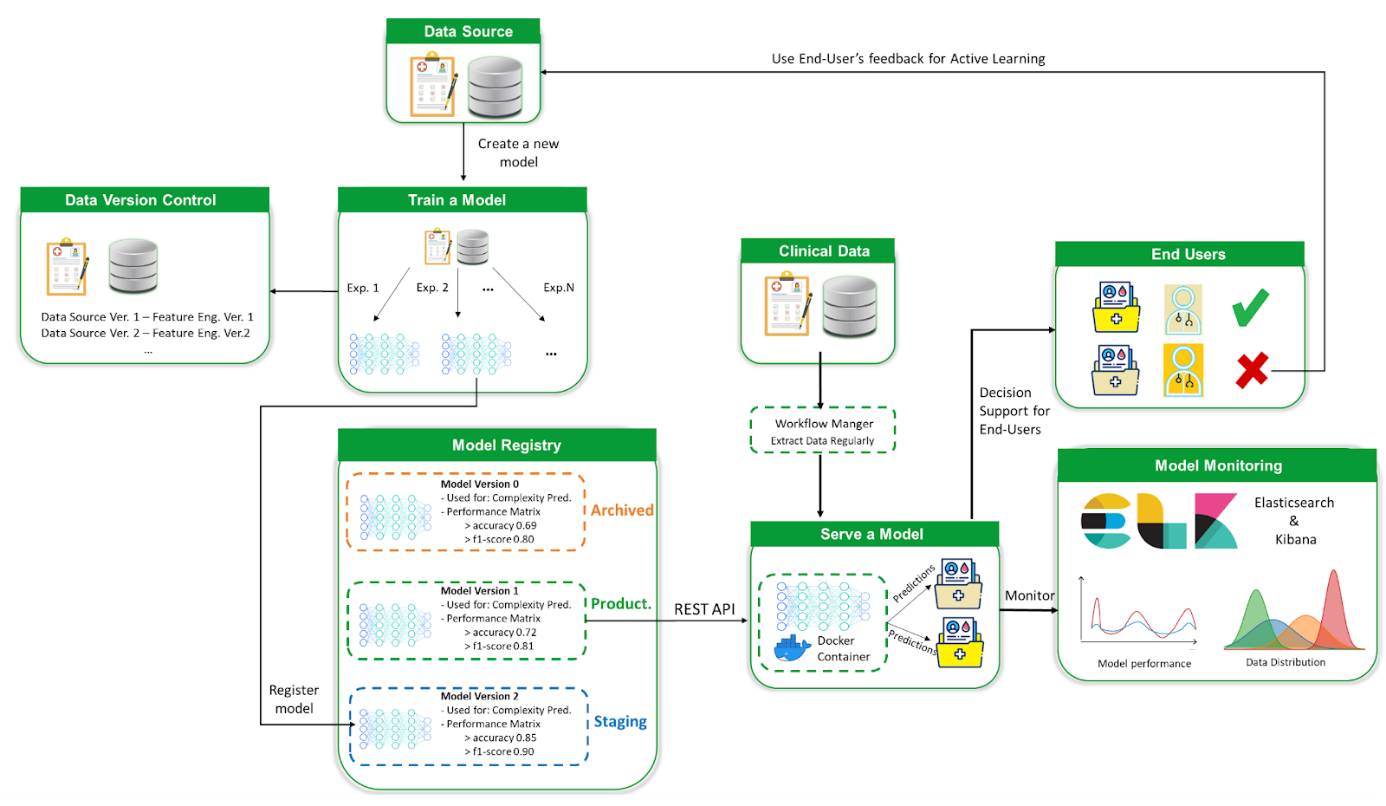


Figure S13 Overview of the integrated model with MLOps infrastructure.

The model will be integrated to our current coding system with a MLOps infrastructure shown in Figure 2. In the model creation phase, data is extracted from the clinical data warehouse (Data Source). Data preprocess and feature engineering are performed and versioned using Data Version Control tools. The selected best model will be stored in a Model Registry. The Model Registry stores different versions of models for an application. If the new model is not used in the production environment yet, it will be tagged with “Staging” status. Models prior to the production version are tagged with “Archived” status, which can be used as backup models in case the model used in the production environment fails. A workflow manager extracts clinical data on a regular basis and pushes the data to the model in production. The model predicts the complexities of medical cases and sends the predictions to the end-user application. The prediction is shown on the UI of the end-user application, and can be corrected by the end-users. The corrections are then sent back to the clinical data warehouse (Data Source) to be used for model re-training. To make sure the model’s performance maintains good quality, we also implement a Model Monitoring platform. The Model Monitoring platform will monitor the distribution of the data fed into the model, as well as the distribution of model predictions. In case the model performance is impaired, the data scientists can roll back to one previously archived model for production, and work on debugging the failed model.

1. Model Performance Compare

Table 1. Comparison of performance using different models and input features on 5-fold cross-validated training dataset (1751 cases), and the best model performance on the test set (309 cases).

| Model  (Features) | Classification  macro-f1 | Classification  accuracy | Regression  r^2^ | Classification^g^  r^2^ | Regression^g^  macro-f1 | Regression^g^  accuracy |
| --- | --- | --- | --- | --- | --- | --- |
|  |  |  |  |  |  |  |
| Majority Vote^f^ | 0.17 | 0.55 | -0.01 | - | - | - |
| Average service complexity | 0.22 | 0.55 | 0.01 |  |  |  |
| Logistic Regression  (TF-IDF) | 0.32 | 0.60 | - | 0.09 | - | - |
| Logistic Regression  (FlauBERT) | 0.42 | 0.57 | - | - | - | - |
| Fine-tuned  FlauBERT (only text) | 0.47 | 0.53 |  |  |  |  |
| Logistic Regression  (Metadata) | 0.41 | 0.47 | -0.4 | - | - | - |
| Logistic Regression  (TF-IDF + Metadata) | 0.48 | 0.62 | 0.23 | - | - | - |
| Gradient Boosted Trees  (TF-IDF) | 0.45 | 0.62 | 0.26 | 0.17 | 0.47 | 0.62 |
| Gradient Boosted Trees  (Metadata) | 0.46 | 0.61 | 0.15 | 0.07 | 0.46 | 0.59 |
| Gradient Boosted Trees  (TF-IDF + Metadata) | 0.51 | 0.59 | 0.30 | 0.03 | 0.49 | 0.63 |
| fastText  (customized embedding) | 0.47 | 0.57 | - | -0.01 | - | - |
|  |  |  |  |  |  |  |
| Test Set Performance | | | | | | |
|  |  |  |  |  |  |  |
| Gradient Boosted Trees  (TF-IDF + Metadata) | 0.46 | 0.58 |  |  |  |  |

^f^ *Majority Vote: always predicts the majority class (in our case, complexity 2), serves as a baseline for model prediction performance.*

*^g^ Gray results are only for comparison between classification and regression models.*

1. Out-of-vocabulary (OOV) analysis

The word2vec word embedding trained on the corpus from our clinical data warehouse contains a vocabulary of 225,700 words. The original corpus was processed by stripping accents and keeping only tokens made of alphabetic characters. We applied the same preprocessing to our text (2060 cases) for training. As it is a bag-of-word model, we also removed stopwords and one-character words. Following this, we end up with 1.56M tokens in total, and 38k unique ones. Out of these, only 8% (124k tokens, and 2.7k unique ones) of the tokens are OOV. We think this low percentage explains the absence of improvement when adding support for subwords.

For our 2060 selected cases, the OOV ratio is about 10%. The distribution of OOV ratio among the 2060 cases is shown below in Figure 3. In our current model, we did not provide a solution for the OOV issue because we do not have enough data to train the word embeddings again. We plan to perform the word embedding and tf-idf retraining once per year instead of retraining more frequently. With this approach, we are hoping to improve the model performance and reduce the impact of the OOV issue.
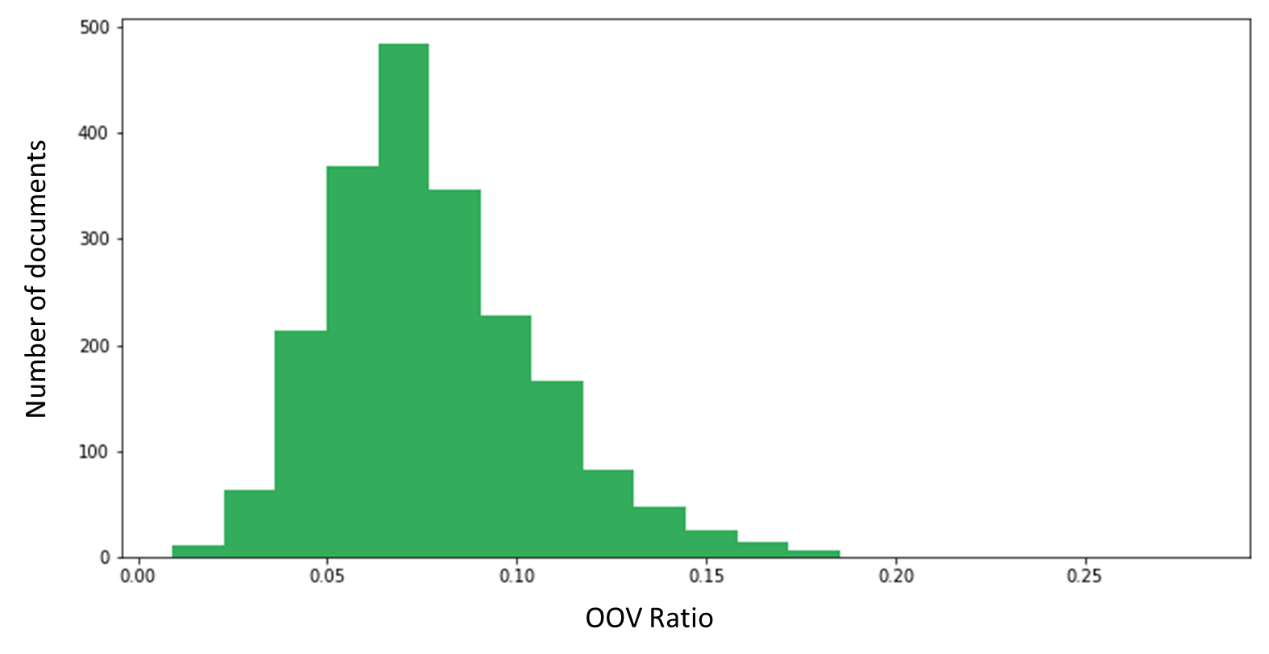


Figure S3 OOV ratio distribution over the 2060 selected cases.

The OOV ratio of the documents distributed in each level of complexity is presented in Table 1.

1. Fine-tune the transformer model for text classification

We experimented with the Flaubert and Camembert models. We carried out model selection, considering among other things: transfer learning settings (fine-tuning the whole model or only last encoder laye), cased vs uncased (when available), loss function (using e.g., a custom cross-entropy to counter class imbalance) as well as different weight decays and other hyperparameters. We detailed our experiments and analysis below.

Firstly, we used the Huggingface transformers library, this library together with the Huggingface datasets library provides convenient wrappers for tokenization, handling truncation and padding. For training, we use <Model>ForSequenceClassification, TrainingArgument and Trainer high-level APIs, which are well suited for our use-case. From there, we run multiple experiments:

1. Freeze pretrained layers: In our very first attempt (prior to this review), we left the entire model trainable (default settings), which overfits strongly from the start as expected. The training loss was going down fast while the validation loss mostly went up. To overcome this problem we run new experiments by freezing the first 11 encoder’s layers and training the last encoder’s layer and the classification layer. This significantly reduced the overfitting.
2. Pretrained models: Flaubert-base-uncased outperformed Flaubert-base-cased by a fair margin. We also tried fine-tuning Camembert-base [https://arxiv.org/abs/1911.03894] with the same approach . Like others have reported, [https://pubmed.ncbi.nlm.nih.gov/35430035/#:~:text=The%20comparisons%20showed%20that%20FlauBERT,ones%20whatever%20the%20language%20model.], Flaubert outperforms Camembert on a problem using medical data (in our +2-3% absolute macro-f1).
3. Weight classes: We also tried to weight classes by setting a custom cross-entropy loss, assigning lower weights to the most common classes and higher weights to the rarer classes. This slightly helped the results (+3% absolute macro-f1).
4. Weight decay: To attempt to further reduce overfitting, we experimented with weight decay which did not help.
